# B-cell depletion induces a shift in self antigen specific B-cell repertoire and cytokine pattern in patients with bullous pemphigoid

**DOI:** 10.1038/s41598-019-40203-7

**Published:** 2019-03-05

**Authors:** Nicolas Berkani, Pascal Joly, Marie-Laure Golinski, Natacha Colliou, Annick Lim, Anis Larbi, Gaetan Riou, Frederique Caillot, Philippe Bernard, Christophe Bedane, Emmanuel Delaporte, Guillaume Chaby, Anne Dompmartin, Michael Hertl, Sebastien Calbo, Philippe Musette

**Affiliations:** 1 Normandie University, UNIROUEN, INSERM U1234 Rouen, France; 2 Normandie University, UNIROUEN, Rouen University Hospital, Department of Dermatology, French reference center for autoimmune bullous diseases, F76000 Rouen, France; 3 Immunoscope plateform, Pasteur Institute, Paris, France; 4 Singapore Immunology Network (SIgN), Agency for Science, Technology and Research (A*STAR), Singapore, Singapore; 5 Department of Dermatology, Reims University Hospital, Reims, France; 6 Department of Dermatology, Limoges University Hospital, Limoges, France; 7 Department of Dermatology, Lille University Hospital, Lille, France; 8 Department of Dermatology, Amiens University Hospital, Amiens, France; 9 Department of Dermatology, Caen University Hospital, Caen, France; 10 Department of Dermatology and Allergology, Philipps University, Marburg, Germany; 11 INSERM U976, Saint Louis Hospital, Paris, France

**Keywords:** Autoimmunity, Autoimmune diseases

## Abstract

Bullous Pemphigoid is the most common auto-immune bullous skin disease. It is characterized by the production of auto-antibodies directed against 2 proteins of the hemi-desmosome (BP180 and BP230). We assessed the efficacy and mechanisms of action of rituximab, an anti-CD20 monoclonal antibody, in 17 patients with severe and relapsing type of bullous pemphigoid. The phenotype, cytokine gene expression, and rearrangement of BP180-specific B-cell receptor genes were performed over 2 years following treatment. At the end of the study, 5 patients had died, 3 had withdrawn from the study, and 9 patients were in complete remission. The one- and two-year relapse rates were 44.1% (95% Confidence Interval (CI): 21.0–76.0%) and 66.5%, (95% CI: 38.4–91.4%), respectively. Phenotypic analyses confirmed dramatic B-cell depletion, which lasted for 9 to 12 months. The ELISA values of serum anti-BP180 antibodies and the frequency of BP180-specific circulating B cells decreased dramatically following treatment, which paralleled the improvement of skin lesions. During B-cell reconstitution, a polyclonal IgM repertoire appeared and a shift in the rearrangement of the B-cell receptor genes of BP180-specific circulating B cells was observed. Concurrently, we observed a decrease of IL-15, IL-6 and TNFα expressing BP180-specific B cells, and the emergence of IL-10 and IL-1RA-expressing BP180-specific IgM+ B cells in patients in complete remission off therapy, suggesting the functional plasticity of BP180-specific auto-immune B cells after rituximab treatment.

## Introduction

Bullous Pemphigoid (BP) is an auto-antibody mediated blistering skin disease characterized by the production of IgG antibodies directed against two hemi-desmosome proteins namely BP230 and BP180, the latter being considered as the major antigen of BP^1–3^. The binding of auto-antibodies to the immuno-dominant NC16A domain of BP180 leads to activation of complement, and recruitment and activation of eosinophils and neutrophils, that disrupt the basement membrane zone (BMZ), and induce blister formation in the skin^4–6^. Topical or oral corticosteroids are considered the mainstay of treatment for BP^7,8^. However, up to 40% of BP patients relapse during steroid tapering, requiring steroid re-increase or associated immunosuppressive drug usage.

B-cell depletion therapy by rituximab has been demonstrated to be highly effective in the treatment of auto-antibody mediated auto-immune diseases such as pemphigus^9,10^, myasthenia gravis^11^ and auto-immune thrombocytopenia^12^. Rituximab induces profound B-cell depletion and eliminates circulating B cells bearing pathogenic auto-antibodies^10,13^. However, other mechanisms have been suggested to be involved in the long-lasting effect of rituximab. Indeed modification of B-cell repertoire after B-cell depletion may explain a shift in the auto-immune response. Moreover, cytokine expression by B cells could be a mechanism of disease control by modifying the balance between pro- and anti-inflammatory cytokines produced by B cells^14,15^. However, the impact of B-cell depletion on the balance between B-cell subpopulations and its regulation on the pathogenesis of autoimmune diseases remain largely unknown. Until now, the immunological effect of rituximab has been studied on total circulating B cells, but this approach does not reflect B cell depletion effects on auto-reactive antigen-specific B-cell phenotype.

In order to assess the effect of rituximab on specific B-cell subpopulations and the molecular mechanisms involved in complete remission and relapse, we studied the BP180-specific auto-immune response in 17 patients with relapsing type of BP who were treated with one cycle of rituximab. Autoimmune B cells were analyzed after single cell sorting without *ex-vivo* stimulation. B cell receptor and cytokine genes of BP180-specific B cells were studied both in remitted patients and in patients who relapsed after rituximab therapy.

## Results

### Clinical outcome

**Figure 1:**
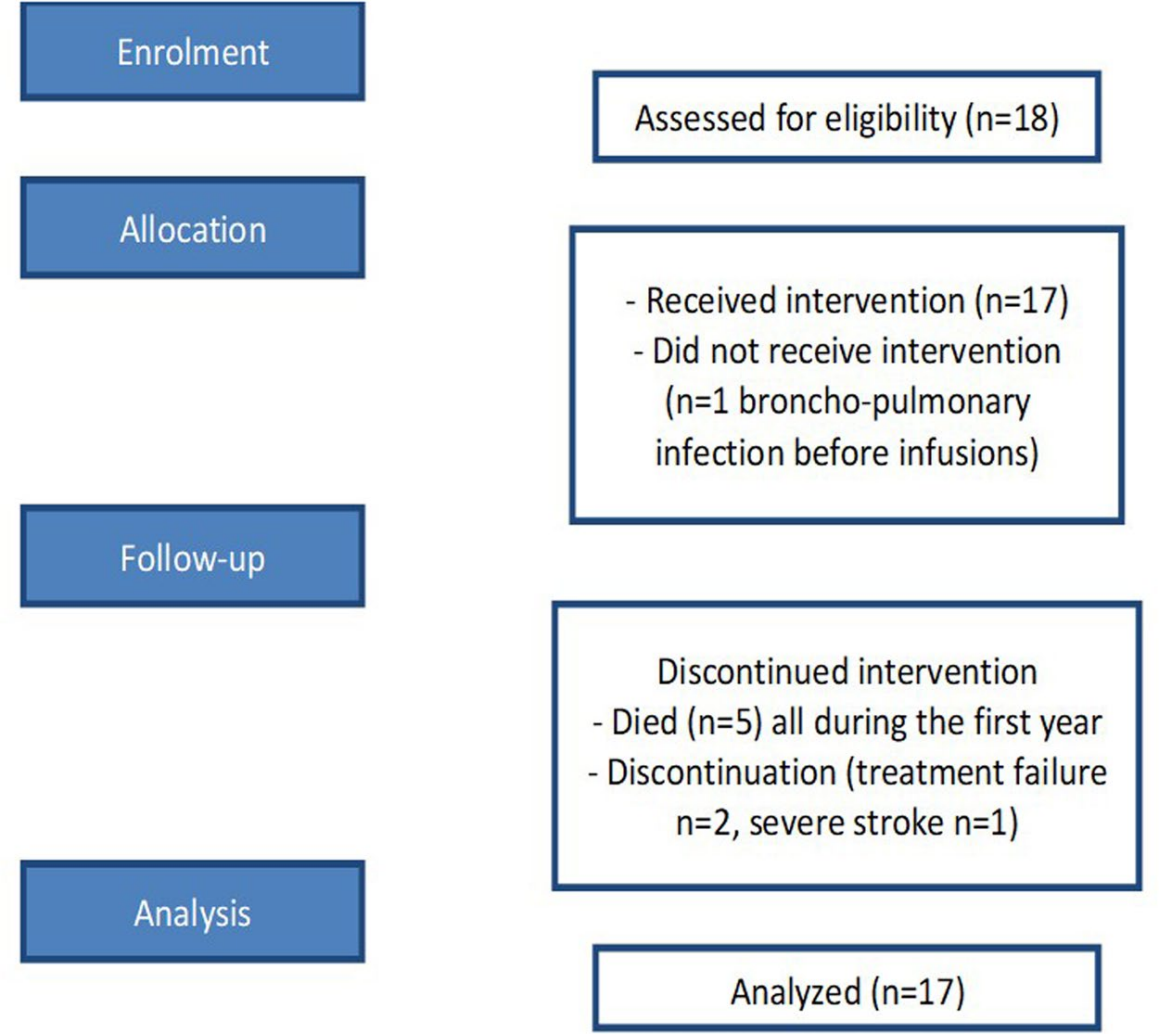
Flow diagram of the clinical trial.

Eighteen patients with relapsing BP were enrolled, but only 17 were treated with rituximab, since one patient had a pneumonia episode the day before rituximab infusion. This patient withdrew from the study before receiving rituximab and was excluded from further analysis. A flow diagram of the trial is shown in Fig. 1. Patients’ main characteristics are described in Supplementary Table 1. The mean age of patients was 77.7 ± 10.9 years. Mean duration of BP before rituximab treatment was 26.7 ± 12.7 months. The mean number of new blisters per day at time of inclusion was 31.9 ± 43.3. All patients achieved disease control at month (M)3 after rituximab treatment. Two patients withdrew from the study on day (D)270 and D540 for treatment failure, and one patient for a stroke which occurred at the first rituximab infusion. Severe treatment adverse events included five deaths which occurred during the first year of the trial caused by general status alteration, n = 2; acute respiratory failure, n = 1; cardiac failure, n = 1; gastro-intestinal bleeding, n = 1, and two pneumonias which occurred at D10 and D270. Of the 9 patients who completed the study, 2 achieved complete remission off-therapy (CRoffT) at M24 without any relapse during the study, and 7 were in complete remission on minimal therapy (CRMT) at M24 still receiving a low dose of topical corticosteroids after the occurrence of relapses. When patients relapsed topical corticosteroids were transiently increased until control of the disease. Five patients relapsed during the first year corresponding to a one-year relapse rate of 44.1% (95% CI: 21.0–76.0%) and 7 patients had relapsed after 2-years of follow-up corresponding to a relapse rate of 66.5%, (95% CI: 38.4–91.4%), respectively.

### Auto-antibody follow-up

**Figure 2:**
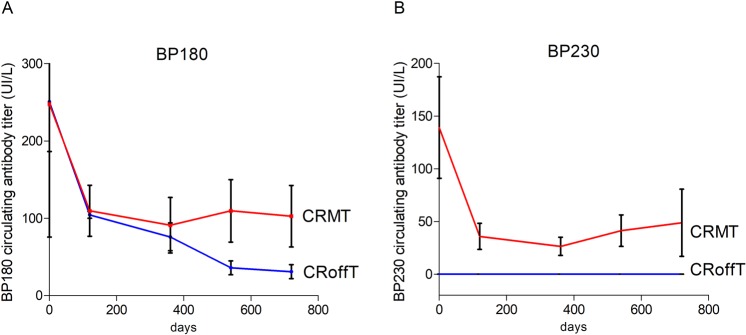
Evolution of anti-BP180 (panel A) and anti-BP230 (panel B) antibody ELISA values in sera from BP patients after Rituximab therapy. Blue lines correspond to patients who achieved complete remission off therapy (CRoffT). Red lines correspond to patients who were in complete remission on minimal therapy (CRMT).

We first investigated the evolution of serum anti-BP180 and anti-BP230 auto-antibodies following rituximab treatment (Fig. 2). Before treatment, 15/17 and 5/17 patients had anti-BP180 and anti-BP230 auto-antibodies, respectively. During the 6-month period after rituximab infusions, all but two patient had a major decrease of anti-BP180 and anti-BP230 auto-antibodies from mean initial ELISA values of 248.5 ± 54.9 IU/L for anti-BP180 and 138.8 ± 48.2 IU/L for anti-BP230 antibodies at D0 to 86.2 ± 23.4 IU/L and 37.0 ± 18.2 IU/L, respectively at M6. A disappearance (ELISA values < 20 IU/L) of anti-BP180 and anti-BP230 auto-antibodies at M6 was observed in 8 and 2 cases, respectively. The two patients who had no major decrease of anti-BP180 and anti-BP230 auto-antibodies after the initial cycle of rituximab, relapsed at day 90 and Day 120. A re-increase of anti-BP180 and/or anti-BP230 antibody ELISA values from M6 to the end of the study was observed in 6 of 8 relapsing patients, corresponding to mean BP180 and BP230 ELISA values of 102.9 ± 39.7 IU/L and 39.0 ± 26.5 IU/L, respectively (Fig. 2). In contrast, the two patients who achieved CRoffT and did not further relapse had a rapid, dramatic and long-lasting decrease of anti-BP180 (Fig. 2A) whereas anti-BP230 antibodies were undetectable up to 2 years after treatment (anti-BP180 and anti-BP230 ELISA values = 31 ± 9 UI/L, and 0 UI/L, at M24, respectively (Fig. 2B). Whereas patients who achieved CRMT presented a slight re-increase of anti-BP180 and anti BP230 after 1 year (Fig. 2).

### Phenotype of B-cell subpopulations

**Figure 3:**
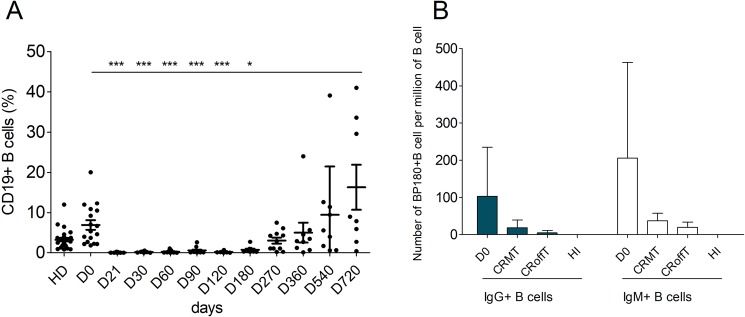
Evolution of blood B cells in BP patients treated with rituximab. Panel A: Percentage of total CD19+ B cells (Kruskal-Wallis test Dunn’s comparison relative to day 0). Panel B: Number of BP180-specific B cell IgM+ (white bars) or IgG+ (blue bars) in BP patients at day 0, in BP patients in complete remission off therapy (CRoffT) or on minimal therapy (CRMT) at Month 24, and in healthy individuals (HI) (Mann Whitney T-Test).

Flow cytometry phenotype analysis of B-cell subpopulations was performed at days 0, 21, 30, 60, 90, 120, 270, 360, 540, 720 throughout the trial. A major depletion in total B-cell population was observed from baseline to months 9–12 in all patients after rituximab infusions (Fig. 3A). Two patients (BP01 and BP03) had a more prolonged B-cell depletion, which lasted for 18 and 24 months, respectively.

Phenotype analysis of circulating B-cell subpopulations showed a long-lasting reversal of the balance between naive and memory B-lymphocytes (Fig. 1). Indeed, the mean percentage of naive B cells in patients’ blood 2 years after rituximab treatment was significantly higher than at D0 (84.85 ± 5.317% versus 63.04 ± 6.01%, p = 0.0313; Fig. S1). Conversely, the mean percentage of memory B cells decreased from 26.94 ± 6.01% at D0 to 15.15 ± 5.317% at M24 (p = 0.0313, Fig. S1). Reconstitution of B cell populations after treatment was characterized by the emergence of transitional B cells from 1.57 ± 1.10% at D0 to 6.44 ± 2.01% at M9, which remained elevated up to M24 at 5.11 ± 1.95% (Fig. S1). Interestingly, the number of IL-10-producing regulatory-B-cells remained stable at 7.34 ± 3.28% before rituximab treatment and 7.76 ± 3.98% at M24 (Fig. S1).

The frequency of BP180-specific B cells was determined by flow cytometry, with a HIS-tagged recombinant immuno-dominant NC16A peptide (Fig. 3B). Rituximab induced a dramatic and long-lasting decrease in BP180-specific IgM+ and IgG+ B cells from 205.9 ± 257.2 and 102.9 ± 132.3 per million CD19+ B cells at baseline, to 31.6 ± 13.9 and 16.0 ± 12.4 per million CD19+ B cells at M24, respectively, (p = 0.226 and p = 0.082), corresponding to a 5-fold decrease in BP180-specific B lymphocytes. Notably, the 2 patients in CRoffT at M24 had a tendency to present a lower number of IgM+ and IgG+ BP180-specific B cells than patients in CRMT IgM+: 17.67 ± 19.66 versus 37.57 ± 21.32 per million B cells (*p* NS); IgG+, 3.33 ± 5.77 versus 21.43 ± 21.16 per million B cells (*p* NS) (Fig. 3B). After D0 BP180-specific B cells were only measurable when B cell compartment is reconstituted at M24. Because during total B cell depletion circulating anti BP180-specific B cells completely disappeared. At M24 BP180-specific B cells reappeared with the B cell reconstitution but with a lower frequency compared to D0. No difference in BP180-specific B cells frequency was found between CRMT and CRoffT patients (Fig. 3B).

### B-cell repertoire

In order to further understand the effects of rituximab on B-cell compartment, we investigated B-cell repertoire diversity. Using immunoscope, we first analyzed the global repertoire of whole circulating IgG+ and IgM+ B cells in 4 patients before and after rituximab treatment, and in 2 healthy individuals (HI). The four patients analyzed at D0 were patients BP07, BP08, BP09, BP11 and at D540 or D720 patients BP07, BP08, BP13, BP06. One patient (BP06) did not relapse during the trial and finished in complete remission, 3 patients did relapse but finished the study in minimal treatment. Before rituximab treatment, an oligoclonal bias was observed on the IgM+ and IgG+ B cells of BP patients, suggesting a restricted repertoire. After rituximab, patients’ B cells recovered a Gaussian polyclonal IgM repertoire. In contrast, some expansions were still observed in the IgG repertoire of all 4 patients, suggesting the persistence of oligoclonal expansions. No difference was found in patients with or without relapses (Data not shown).

**Figure 4:**
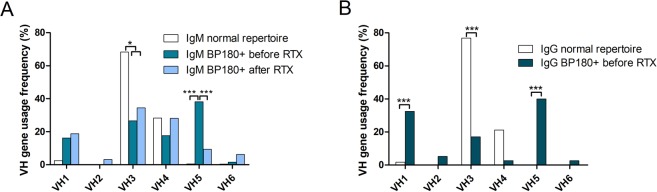
Representation of VH family frequency usage by BP180-specific IgM (panel A) or IgG (panel B) circulating B cells from BP patients before (medium-blue column) or after (light-blue column) rituximab treatment at different time points D360, D540 and D720, compared with non-autoreactive B cells from healthy individuals (white column) (Fisher exact test).

We then focused on the repertoire of BP180-specific autoimmune B cells by sequencing the B-cell receptor (BCR) H-CDR3 before and after rituximab treatment. A more frequent use of the VH5 gene family by BP180+ IgM+ B cells was observed at baseline relative to after rituximab treatment (37.9 ± 11.9% versus 6.1 ± 8.3%, respectively; p < 0.0001). (Fig. 4A). After rituximab treatment, the repertoire of the BCR heavy chain of IgM+ BP180-specific B cells recovered a standard distribution of all VH families (Fig. 4A).

The repertoire of BP180+ IgG+ B cells could only be analyzed at baseline, since insufficient numbers of IgG sequences were obtained after rituximab treatment, due to the persistent B-cell lymphopenia and extremely low frequency of circulating BP180-specific IgG+ B cells after rituximab treatment. A preferential use of VH1 and VH5 gene families was observed in BP180-specific IgG+ B cells from patients before rituximab treatment as compared with the global repertoire of HI (40 ± 15.5% versus 1.7 ± 1.6%, p < 0.0001; and 32.5 ± 14.8% versus 0.7 ± 0.6%, p < 0.0001, respectively) (Fig. 4B). Conversely, the VH3 gene family was less frequently used by BP180-specific IgG+ B cells from BP patients compared with the global repertoire of HI (17.1 ± 11.8% versus 76.9 ± 5.3%, p < 0.0001).

Interestingly, the analysis of amino-acid composition of IgG+ BP180-specific CDR3 collected from BP patients at baseline showed redundant motifs by the use of a Glycine in position 114 (Kabat numbering scheme) in 65 ± 15.1% of sequences.

Finally, after treatment, patients recovered a Gaussian polyclonal global repertoire, suggesting that rituximab can induce changes in the BP180-specific B-cell repertoire as evidenced by a change in VH usage.

### cytokine secretion profile of BP180-specific B cells

In order to characterize the cytokines produced by BP180-specific auto-reactive B cells, we analyzed the expression of 25 cytokines and 5 housekeeping genes using high throughput quantitative polymerase chain reaction (qPCR) in BP180-specific B cells from BP patients collected before and after rituximab treatment (Table S2). We analyzed a total of 364 single B cells including 224 BP180-specific B cells collected at different time points (D0, D360, D540 and D720) from 10 BP patients either in CRoffT or in CRMT after rituximab treatment. One hundred BP180-negative B cells were collected from the same BP patients as negative control experiments, and 40 B cells from 2 healthy individuals. We first evidenced that the frequencies of cytokine genes expressed in BP180-negative B cells from BP patients did not differ between samples collected before or after rituximab treatment, whereas BAFF was over expressed on BP180-negative B cells in patients presenting CR compared to D0 and healthy controls (Fig. S2).

Analysis of pro-inflammatory cytokine gene expression by BP180-specific B cells showed a large decrease in the frequency of IL15 and IL6 expressing B cells after rituximab treatment compared to D0. Indeed, the IL-15 gene was the most frequently expressed cytokine by BP180-specific B cells ranging before treatment from 29.3 ± 10.5% to 15.9 ± 8.7% after rituximab treatment (p = 0.0203). IL-6 expressing B cells represented 10.9 ± 10.5% of BP180-specific B cells before treatment and only 0.8 ± 8.7% (p = 0.0007) after rituximab treatment. Interestingly, only IL-15 and IL-6 expressing B cells seemed to be impacted by rituximab treatment. The frequency of BP180-specific B cells expressing TNFα, TNFβ, TRAIL and IL1β was variable and did not significantly change after rituximab treatment.

**Figure 5:**
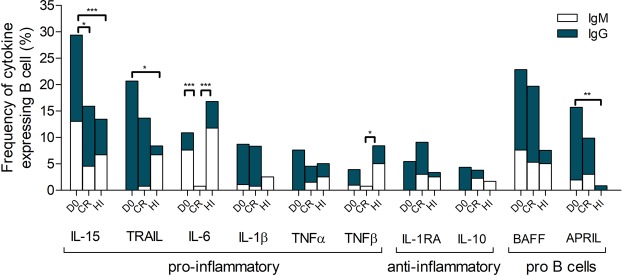
Frequency of pro-inflammatory cytokines, anti-inflammatory cytokines and B cell stimulatory cytokines expressed by BP180-specific IgM+ and IgG+ B cells in CR patients before (D0) and after rituximab treatment. CR = CRMT+ CRoffT and Healthy Individuals (HI) (Fisher exact test).

Regarding anti-inflammatory cytokines, the frequency of IL-1RA and IL-10 expressing BP180-specific B cells was measured at a low level after and before rituximab treatment (IL-1RA: 5.4 ± 10.5% at D0 versus 9.1 ± 8.7% at CR; p = 0.2251; IL-10: 4.4 ± 10.5% at D0 versus 3.8 ± 8.7% at CR; p = 1). Interestingly, BP180-specific IgM+ B cells expressing IL-10 and IL-1RA were only found in CR patients and were undetectable before rituximab treatment (Fig. 5).

Finally, cytokine mRNA expression analysis revealed a shift in the frequency of auto-reactive B-cell populations expressing cytokine genes. Rituximab induced the decrease of pro-inflammatory cytokine-expressing BP180-specific B cells namely IL-15 and IL-6 and promoted the expression of anti-inflammatory cytokines including IL10 and IL1RA in auto-reactive BP180-specific IgM+ B cells in CR patients. Interestingly in CR patients, TNFβ was decreased compared to HI, whereas TRAIL and APRIL were increased in bullous pemphigoid patients irrespectively of their clinical status compared to HI.

## Discussion

We report the efficacy of rituximab in the treatment of severe types of BP, and found that clinical remission after rituximab was associated with a shift in the B cell receptor gene usage and cytokine pattern expression of BP180-specific B cells which reappeared after rituximab B cell depletion. Despite the fact that all patients achieved disease control after initial treatment, rituximab seems less effective in BP patients than in pemphigus patients, since 7 of 17 patients (41%) were in complete remission on minimal therapy and only 2 patients (12%) achieved CRoffT at M24 as compared with 89% of CR off therapy in pemphigus patients^9,10^. Moreover, 2 BP patients withdrew from the study for treatment failure. However, it should be highlighted that the BP patients included in the present trial had a particularly severe disease, since all of them had previously relapsed twice before inclusion despite previous topical and systemic therapies. In addition, the tolerance of rituximab in BP patients seems poorer than in pemphigus patients with 7 severe adverse events including 5 deaths (29%), which is however in accordance with the 2-year mortality rate of BP patients, and likely related to their old age and associated medical conditions^16,17^. The tolerance in our series was slightly poorer than that reported by Ahmed *et al*. in a retrospective series in the US, which is in accordance with the younger age by almost 10 years of the BP patients in this US series compared to the present trial (68.2 years versus 77.7 years)^18^.

Immunological analyses first showed that only the two patients who achieved CRoffT had a dramatic and long-lasting decrease of serum anti-BP180 antibodies after rituximab therapy, which is in accordance with the fact that many patients further relapsed after the initial cycle of rituximab and had to keep a minimal CS therapy to maintain the remission of BP lesions. In all patients, we observed the re-emergence of transitional B cells and a reversal of the balance between naive and memory B cells after B-cell reconstitution; this total B-cell phenotype shift may be instrumental for controlling autoimmune response, by stimulating the production of suppressive or regulating cytokines^15,19–25^.

To further analyze the mechanisms involved in long-lasting CR and relapses after rituximab B-cell depletion, we measured the expression of a panel of 25 cytokine genes in a BP180-specific B-cell population at the single cell level. To assess the *in vivo* function of BP180-specific circulating B cells, these analyses were performed by single cell qPCR, directly after single cell sorting with no *in vitro* activation or stimulation before qPCR experiments. Interestingly, no change in cytokine expression by BP180-negative B cells was observed between samples collected at baseline or after rituximab treatment. The cytokine expression in BP180-negative B-cell populations was similar to that observed in whole blood B cells from HI, whereas BAFF was over expressed in CR patients. In contrast, significant changes in the frequency of cytokine expressing B cells were evidenced in BP180-specific B-cell populations. First, we observed a major decrease of the expression of IL-15 and IL-6 by BP180-specific B cells after rituximab treatment, relative to baseline. Interestingly, the expression of anti-inflammatory cytokines including IL-10 and IL-1RA by IgM+ BP180-specific B cells was only detected in the two patients in CRoffT. Altogether, the increased frequency of IgM+ IL-10 and IL-1RA, and the concomitant decrease in the pro-inflammatory IL-15 and IL-6 expressing B cells suggest that this cytokine shift might be involved in the long-term remission of BP patients after rituximab treatment^25–27^.

IL-15 was the most frequently expressed cytokine gene by BP180-specific B cells before rituximab treatment. The role of IL-15 in the pathogenesis of BP has never been evidenced. It has been hypothesized in a model of human eosinophilic esophagitis that IL-15 could stimulate helper T cells to produce eosinophil-selective chemoattractants^28,29^. Indeed, the binding of IL-15 to its receptor IL-15R on helper T cells has been demonstrated to activate STAT5, thus inducing the secretion of IL-5, IL-13, eotaxins 1 to 3, and Th2 cytokines by T cells^30^. As a consequence IL-15 may be a cytokine that plays a key role in the eosinophilia found in BP patients.

Despite the fact that rituximab induced a major decrease in BP180-specific IgG+ and IgM+ B cells in BP patients, these autoimmune B cells remained detectable after B cell reconstitution, even in patients in complete remission. To further disentangle these results, we sequenced the CDR3 of the BCR heavy chain of BP180-specific B cells before and after treatment. At baseline, both BP180-specific IgM+ and IgG+ B cells preferentially used the VH5 gene family, suggesting a switch from VH5 BP180-specific IgM+ B cells to pathogenic VH5 IgG+ B cells. Due to the extremely low number of BP180-specific IgG+ B cells collected after rituximab treatment, we were only able to study the evolution of BP180-specific IgM+ B cells. Rituximab treatment strongly impacted the autoimmune selection of BP180-specific IgM+ B cells, which mainly used the VH3 and VH4 gene families after B cell reconstitution, which is in accordance with the Gaussian repertoire of total B cells. Previous observations in rheumatoid arthritis and peripheral nervous system autoimmune diseases have suggested that variations in VH usage and isotopic switch blockade might be involved in the long-term efficacy of rituximab^31,32^. Interestingly 65% of BP180-specific IgG H-CDR3 sequences collected from patients before rituximab exhibited a glycine in position 114, which suggests a selection pressure mechanism on B cells resulting in N-addition or mutations to promote a glycine in position 114. Interestingly, 2 of the 3 anti-BP180 human monoclonal antibodies which have been sequenced in the literature^33^ also exhibit a glycine in position 114 on H-CDR3, which suggests that this might correspond to one mechanism of selection based on pattern recognition of pre-pathogenic BP180-specific IgM+ B cells to become pathogenic IgG+ B cells^33^.

Our study presents some limitations. First, it was an exploratory open series, which was performed on a limited number of BP patients. Despite restrictive inclusion criteria certain patients died or dropped out the study, which not allow assessment of their immune response up to M24.

The major strength of this study is that it allowed for the first time the analysis of the cytokine gene expression of BP180-specific B cells without *ex-vivo* stimulation. Conversely, circulating autoreactive B cells may not reflect the total population of anti-BP180-specific B cells, in particular those which are located in the spleen and bone marrow^34^.

In addition to previous observations showing the role of the balance between naïve and memory B cells in the long-term remission induced by rituximab treatment, our study shows that rituximab induces a shift in VH gene family usage and the expression of anti-inflammatory cytokines by circulating antigen-specific B cells after B-cell reconstitution. This plasticity of the autoimmune B-cell compartment and the disappearance of pro-inflammatory antigen-specific B cells likely play a major role in the long-lasting clinical remission induced by rituximab.

## Materials and Methods

### Study design

Eighteen BP patients aged between 18 and 85 years with a Karnowsky score ≥50% were included in this prospective, non-randomized, open label, multicenter clinical trial. Only 17 of these 18 patients were assessed for the efficacy and tolerance of a single cycle of rituximab in the treatment of recalcitrant and relapsing types of BP since one patient had a pneumonia episode the day before rituximab infusion. All patients had previously experienced at least 2 relapses under oral or topical corticosteroid treatment. Treatment consisted of two intravenous (IV) infusions of rituximab 1000 mg administered at day (D)0 and D15 in all patients. In addition to rituximab, patients were initially treated with topical applications of clobetasol propionate cream to rapidly achieve control of BP lesions. Patients with a moderate type of BP (defined as the occurrence of fewer than 10 new blisters per day) were initially treated with 20 g per day of clobetasol until disease control, and those with extensive BP (defined as the occurrence of 10 or more new blisters per day) were treated with 30 g of clobetasol per day. Topical CS doses were tapered 15 days after disease control achievement and stopped 2 months later. Included patients did not receive oral corticosteroids. Study visits were scheduled weekly during the first month of treatment and then monthly until month 24.

The primary endpoint was the rate of patients who achieved complete remission off therapy (CRoffT) after a single cycle of rituximab, and did not relapse until month (M)24 after rituximab treatment. According to the consensus statement^35^, CRoffT was defined as the absence of new or established lesions while the patient was off all BP therapy for at least two months. Secondary endpoints were: (i) the rate of patients who achieved control of BP lesions 3 months after rituximab treatment, (ii) the rate of patients in complete remission on minimal therapy (CRMT) at M24 (still receiving a prednisone dose <0.1 mg/kg/day, or clobetasol propionate cream <20 g per week for at least two months), (iii) the rate of relapses during the study (defined as the appearance of 3 or more new lesions a month or at least one large (>10 cm diameter) eczematous lesion or urticarial plaque that did not heal within 1 week in a patient who had achieved disease control), and (iv) the number of severe treatment adverse events including death. Complete remission (CR) included patients with CRoffT and CRMT.

### Study approval

This study was approved by the Ethics Committee of the French North West area I. It was registered 06/09/2007 and referenced in ClinicalTrials.gov (number NCT00525616). Written informed consent was obtained from each patient with BP for collection of blood samples. All the methods were performed in accordance with the relevant guidelines and regulations.

### Auto-antibody serum concentrations

Serum concentrations of IgG antibodies against BP180 and BP230 were measured with Bullous Pemphigoid (BP) ELISA tests with 1:100 diluted serums following manufacturer’s protocol (EUROIMMUN Medizinische Labordiagnostika AG).

### Phenotypic analysis

The phenotype of peripheral blood mononuclear cells (PBMCs) was determined by six-color flow cytometry with murine monoclonal antibodies (mAbs) against CD3, CD4, CD8, CD5, CD19, CD20, CD21, CD22, CD23, CD24, CD27, CD38, CD56, and CD86 (Beckman Coulter and BD Biosciences). PBMCs were collected from BP patients before and after rituximab treatment, and from 20 elderly healthy individuals (HI).

### IL-10 regulatory B cell analysis

Purified B cells (5 × 10^5^) were cultured for 48 hours with CpG-B (3 mg/ml) and anti-human IgG+IgA+IgM (anti-Ig) antibody (Jackson Immuno Research) (20 mg/ml) in 1 ml of complete medium in 24-well flat-bottom tissue culture plates. Cells were first stained with anti-CD19, anti-CD24, anti-CD27, anti-CD38, and anti-CD5 mAb, and then fixed and permeabilized, followed by intracellular staining with anti-human IL-10 mAb (B-T10) or mouse IgG1 isotype control (MiltenyiBiotec). All assays were carried out with duplicate samples.

### Anti-BP180 B cell analysis and sorting

Purified B cells collected from BP patients before and after rituximab treatment, and from two HI were incubated for 1 hour at room temperature with histidine-tagged recombinant BP180, 40 µg/ml. After washing, anti-histidine coupled with phycoerythrin (R&D Systems) was used to identify BP180-stained cells. B cells were characterized with anti-human CD19 and anti-human IgG and IgM antibodies (BD Biosciences). The number of BP180-specific B cells per million was then determined.

### CDR3-H amplification and sequences

cDNA was synthesized in a total volume of 14 μl/well in the original 96-well sorting plate. Total RNA from single cells was reverse transcribed in nuclease-free water using 150 ng random hexamer primer (pd(N)6, GE Healthcare), 0.5 μl of 10 mM each nucleotide dNTP-Mix (Invitrogen), 1 μl 0.1 M DTT (Invitrogen), 0.5% v/v NP40, 4 U RNAsin® (Promega), 6 U Prime RNAse Inhibitor™ (Eppendorf) and 50 U Superscript® III reverse transcriptase (Invitrogen). Reverse transcription (RT) reaction was performed at 42 °C for 10 min, 25 °C for 10 min, 50 °C for 60 min and 94 °C for 5 min. cDNA was stored at −20 °C. IgH gene transcripts were amplified by nested polymerase chain reaction (PCR) starting from 3.5 μl of cDNA as template. All PCR reactions were performed in 96-well plates in a total volume of 40 μl per well containing 20 nM primer mix (Table 1), 300 nM each dNTP (Invitrogen) and 1,2 U HotStar® Taq DNA polymerase (Qiagen). All primers were stored in small aliquots to avoid repeated freezing and thawing and all PCRs were performed with nuclease-free water. All nested PCR reactions with primer mix were performed with 3.5 μl of unpurified first PCR product (Table 1). Each round of PCR was performed for 50 cycles at 94 °C for 30 s, 58 °C for 30 s, and 72 °C for 55 s. PCR was checked for amplification in agarose gel 1.5% and revealed with BET and 2 µL of second PCR products. Positive PCR products were purified with Nucleospin Gel and PCR cleanup (Macherey Nagel®) and sequencing reactions were done with selected primers (Table 1) and Big Dye Terminator V3.1 cycle sequencing Kit (AB®) following manual instructions. Sequencing files were analyzed with SeqScanner software (AB®) and blasted on IMGT.

**Table 1:** Primer sequences for CDR3-H amplification and sequence.

PCR 1	VHL-1	5′	TCACCATGGACTGSACCTGGA
	VHL-2	5′	CCATGGACACACTTTGYCCAC
	VHL-3	5′	TCACCATGGAGTTTGGG
	VHL-4	5′	AGAACATGAAACAYCTGTGGTT
	VHL-5	5′	ATGGGGTCAACCGCCATCCT
	VHL-6	5′	ACAATGTCTGTCTCCTTCCTCA
	Cγ II	5′	GCCAGGGGAAGACSGATG
	Cµ II	5′	CAGGAGACGAGGGGAAAAG
PCR 2	VHF-1	5′	TTGCGGCCGCCAGGTSCAGCTGGTRCAGTC
	VHF-2	5′	TTGCGGCCGCCAGRTCACCTTGAAGGAGTC
	VHF-3	5′	TTGCGGCCGCSAGGTGCAGCTGGTGGAGTC
	VHF-4	5′	TTGCGGCCGCCAGGTGCAGCTGCAGGAGTC
	VHF-5	5′	TTGCGGCCGCGARGTGCAGCTGGTGCAGTC
	VHF-6	5′	TTGCGGCCGCCAGGTACAGCTGCAGCAGTC
	Cγ III	5′	AGGTCTAGAGACSGATGGGCCCTTGGTGGA
	Cµ III	5′	AGGTCTAGAGAAAAGGGTTGGGGCGGATGC

### Cytokine genic expression profile

BP180-specific single B cells were sorted by FACS ARIA III into 96-well plates containing 10 µL Platinum Taq polymerase and SuperScript III reverse transcriptase (Invitrogen), a mixture of Taqman primer-probes at 0.2× concentration specific for the transcripts of interest (Supplementary Table 1) and CellsDirect qRT-PCR buffer (Invitrogen). Immediately following cell sorting, samples were centrifuged, incubated at 55 °C for 10 min, and subjected to 20 cycles of PCR (50 °C 15 min then 95 °C for 15 s for the reverse transcription, followed by 20 cycles of 95 °C 15 s and 60 °C 4 min for amplification). Subsequent preamplified single-cell cDNA was stored at −20 °C until analysis. After ¼ dilution in TE buffer, each cDNA sample was then separated into 48 separate reactions for further qPCR using the BioMark 48.48 dynamic array nanofluidic chip (Fluidigm, Inc.). Briefly, following hydraulic chip priming, 48 preamplified cDNA samples were mixed with a mild detergent loading solution to allow capillary flow, and the samples were added to a 48.48 nanofluidic chip (Fluidigm, Inc.) along with 30 individual Taqman primer-probe mixtures listed in Supplementary Table 1 (Applied Biosystems) specific for individual transcripts of interest, allowing a combination of each sample to mix with each probe in every possible combination (a total of 2,304 reactions). The chip was then thermocycled through 40 cycles and fluorescence in the FAM channel was detected using a CCD camera placed above the chip, normalized by ROX (6-carboxy-X-rhodamine) intensity. 100 CD19+ cells and no-cell were used as positive and negative controls respectively. To limit potentially biased measurement, cells with fewer than 2 expressed genes among the 5 control genes (HPRT1, B2M, GUSB, TUBB and GAPDH) were excluded from the analysis. Data were analyzed using Real Time PCR Analysis software with or without normalization of the Ct value for each gene using GAPDH as calibrator gene. All primers were validated from cDNA of purified B cells stimulated or not by qPCR on Lightcycler (Roche). Sorted cells were derived from frozen PBMC. Ten cytokines were found unexpressed by single B cells including IL-2, IL-5, IL-9, IL-12p40, IL-13, IL-17A, IL-17F, IL-21, IL-27p28 and TGFb2. However, positive control wells containing 100 CD19+ cells showed detectable expression levels of all tested cytokine genes. The amount of RNAs contained in one cell was possibly too low, even after pre-amplification for detection and quantification by Biomark technology, or in any of the BP180-specific cells expressing any of those cytokines. Detected genes demonstrated a homogenous level of expression between single cells shown in ΔCt Fig. S5 calculated with GAPDH as reference gene. No significant differences in cytokine gene expression levels between patient groups were found even if expression levels seemed to be higher at D0; therefore we analyzed qPCR results for frequency of cytokine gene expressing B cells compared between groups.

### Total B-cell repertoire

IgG and IgM repertoire was characterized at the molecular level in B cell PBMCs from four BP patients before treatment, four patients after treatment and two HI. Complementary DNA was prepared from 10 × 10^6^ PBMCs in a RLT lysis buffer (QIAGEN®). VH gene usage and CDR3 analysis were performed with the imunoscope method coupled with real time PCR to provide quantitative information on VH usage^13^.

### Statistical analysis

Statistical comparisons were performed with the non-parametric Kruskal-Wallis Anova test, and the Wilcoxon signed-rank test was used for paired samples. Statistical comparison of the frequency of cytokine secreting cells was performed with Fisher’s exact test. All statistical tests were done using GraphPad Prism Software. A p-value ≤ 0.05 was considered as significant.

## Supplementary information

Supplementary Dataset 1
