# Author Correction: B-cell depletion induces a shift in self antigen specific B-cell repertoire and cytokine pattern in patients with bullous pemphigoid

**DOI:** 10.1038/s41598-019-54421-6

**Published:** 2019-12-09

**Authors:** Nicolas Berkani, Pascal Joly, Marie-Laure Golinski, Natacha Colliou, Annick Lim, Anis Larbi, Gaetan Riou, Frederique Caillot, Philippe Bernard, Christophe Bedane, Emmanuel Delaporte, Guillaume Chaby, Anne Dompmartin, Michael Hertl, Sebastien Calbo, Philippe Musette

**Affiliations:** 1 Normandie University, UNIROUEN, INSERM U1234, Rouen, France; 2 Normandie University, UNIROUEN, Rouen University Hospital, Department of Dermatology, French reference center for autoimmune bullous diseases, F76000 Rouen, France; 3 Immunoscope plateform, Pasteur Institute, Paris, France; 4 Singapore Immunology Network (SIgN), Agency for Science, Technology and Research (A*STAR), Singapore, Singapore; 5 Department of Dermatology, Reims University Hospital, Reims, France; 6 Department of Dermatology, Limoges University Hospital, Limoges, France; 7 Department of Dermatology, Lille University Hospital, Lille, France; 8 Department of Dermatology, Amiens University Hospital, Amiens, France; 9 Department of Dermatology, Caen University Hospital, Caen, France; 10 Department of Dermatology and Allergology, Philipps University, Marburg, Germany; 11 INSERM U976, Saint Louis Hospital, Paris, France

Correction to: *Scientific Reports* 10.1038/s41598-019-40203-7, published online 05 March 2019

The original version of this Article contained errors in the names of the authors Nicolas Berkani, Pascal Joly, Marie-Laure Golinski, Natacha Colliou, Annick Lim, Anis Larbi, Gaetan Riou, Frederique Caillot, Philippe Bernard, Christophe Bedane, Emmanuel Delaporte, Guillaume Chaby, Anne Dompmartin, Michael Hertl, Sebastien Calbo & Philippe Musette which were incorrectly given as Berkani Nicolas, Joly Pascal, Golinski Marie-Laure, Colliou Natacha, Lim Annick, Larbi Anis, Riou Gaetan, Caillot Frederique, Bernard Philippe, Bedane Christophe, Delaporte Emmanuel, Chaby Guillaume, Dompmartin Anne, Hertl Michael, Calbo Sebastien & Musette Philippe respectively.

As a result, the original version of this Article contained errors in the Author Contributions section.

“B.N., J.P., C.S. and M.P. wrote the paper. B.N., G.M.L., C.N., C.F., L.i.A., L.a.A., R.G., H.M., C.S. and P.M. acquired and analyzed the data. B.P., B.C., D.E., C.G. and D.A. included patients.”

now reads:

“N.B, P.J., S.C. and P.M. wrote the paper. N.B., M.L.G., N.C., F.C., A.L.i.., A.L.a., G.R., M.H., S.C. and P.M. acquired and analyzed the data. P.B., C.B., E.D., G.C. and A.D. included patients.”

Additionally, the Supplementary Information File ‘Clean Supple Mat’ was a duplication of ‘Supplementary Dataset 1’

These errors have now been corrected in the PDF and HTML versions of the Article, and in the Supplementary Dataset file.

