# Supplementary material for: B-cell depletion induces a shift in self antigen specific B-cell repertoire and cytokine pattern in patients with bullous pemphigoid
Source: Sci Rep. 2019 Mar 5;9:3525. doi: 10.1038/s41598-019-40203-7 (PMC6401188; doi:10.1038/s41598-019-40203-7)
Supplement: Supplementary file 1 — Supplementary Dataset 1 [file 41598_2019_40203_MOESM1_ESM.doc]

**B-cell depletion induces a shift in self antigen specific B-cell repertoire and cytokine pattern in patients with bullous pemphigoid.**

Nicolas Berkani, Pascal Joly, Marie-Laure Golinski, Natacha Colliou, Annick Lim, Anis Larbi, Gaetan Riou, Frederique Caillot , Philippe Bernard, Christophe Bedane , Emmanuel Delaporte, Guillaume Chaby, Anne Dompmartin, Michael Hertl, Sebastien Calbo and Philippe Musette

**Supplementary Materials:**

Supplementary Figure 1: Evolution of peripheral blood B cell subpopulations in BP patients treated with rituximab. Control B cells were collected from elderly healthy individuals. Panels A,B,C and D show the frequency of CD19^+^CD27^+^ memory B lymphocytes, CD19^+^CD27^-^ naive B lymphocytes, CD24^high^CD38^high^ transitional B lymphocytes and IL10^+^ B lymphocytes, respectively. Patients in complete remission (CR) off therapy are represented by red dots and patients in CR on minimal therapy by black dots. Statistical analysis was performed by non-parametric paired Wilcoxon T-test, *p < 0.05.

Supplementary Figure 2: expression of cytokine genes by BP180- IgM+ and IgG+ circulating B cells from BP patients before (D0) and after rituximab treatment in patients in CR off therapy (CRoffT) and in CR on minimal therapy (CRMT), and from two healthy individuals (HI). Pro-inflammatory cytokines, Anti-inflammatory cytokines and B-cell stimulatory cytokines. Statistical analysis was performed by Fisher exact test, *p < 0.05. CR = (CRMT + CRoffT)


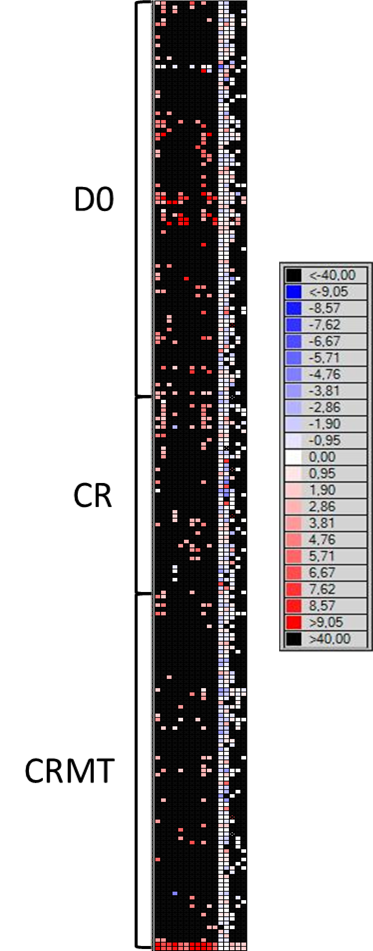


Supplementary Figure 3: Hit Map of BP180-specific B cells from D0, CRoffT and CRMT BP patients showing delta CT mRNA measurement reported to GAPDH CT value. Tested genes from the left to the right IL-15, TRAIL, IL-6, IL-1β, TNFα, TNFβ, IL-1RA, IL-10, BAFF, APRIL, BAFF-R, GAPDH, B2M, TUBB, GUSB and HPRT1.

Table S1: characteristics of patients and clinical status.

| Patient Id | Gender (Male / Female) | Age (years) | Disease  Extent  moderate (M) extensive (E) | Relapses | Adverse events | Clinical  Status at the study end |
| --- | --- | --- | --- | --- | --- | --- |
| BP01 | M | 85 | M | Yes at D600 |  | CR |
| BP02 | F | 59 | M | No | Pneumonia at D10 Death at D60 | Dead |
| BP03 | F | 82 | E | Yes at D150 | Pneumonia at D270 | CR |
| BP04 | M | 77 | M | No | Death at D45D | Dead |
| BP05 | F | 79 | E | No |  | CRoffT |
| BP06 | M | 66 | M | No |  | CR |
| BP07 | F | 82 | M | Yes at D300 |  | CR |
| BP08 | F | 80 | E | Yes at D270 |  | Dropped out of the study (treatment failure D 270) |
| BP09 | F | 82 | M | No | Death at D360 | Dead |
| BP10 | F | 85 | E | No |  | CRoffT |
| BP11 | M | 86 | E | No | Death at D60 | Dead |
| BP12 | F | 79 | M | No | Death at D210 | Dead |
| BP13 | F | 83 | E | Yes at D270 | Leg cellulitis at D21 | CR |
| BP14 | F | 85 | E | No | Stroke at D1 | Dropped out of the study (adverse event ) |
| BP15 (Not included  (pneumonia at D0) | M | 79 |  |  |  |  |
| BP16 | F | 77 | M | Not controlled at D90 |  | Dropped out of the study ( treatment failure D 90) |
| BP17 | M | 45 | E | Yes D120 |  | CR |
| BP18 | M | 81 | M | Yes D570 |  | CR |

Abbreviations: BP, bullous pemphigoid; CR, complete remission; CRoffT, complete remission off therapy

Table S2: Taqman primers used in High Throughput qPCR Biomark analysis.

| **Gene Name** | **Assay ID** |
| --- | --- |
| **Cytokine Genes** |  |
| IL-1β | Hs01555410_m1 |
| IL-1RA | Hs00893626_m1 |
| IL-2 | Hs00174114_m1 |
| IL-5 | Hs01548712_g1 |
| IL-6 | Hs00985639_m1 |
| IL-7 | Hs00174202_m1 |
| IL-9 | Hs00914237_m1 |
| IL-10 | Hs00961622_m1 |
| IL-12A | Hs01073447_m1 |
| IL-12B | Hs01011518_m1 |
| IL-13 | Hs00174379_m1 |
| IL-15 | Hs01003716_m1 |
| IL-17A | Hs00174383_m1 |
| IL-17F | Hs00369400_m1 |
| IFNγ | Hs00989291_m1 |
| TNFα | Hs01113624_g1 |
| LTA | Hs04188773_g1 |
| TNFSF10 | Hs00921974_m1 |
| IL-21 | Hs00222327_m1 |
| TGFβ2 | Hs00234244_m1 |
| TNFSF13B | Hs00198106_m1 |
| TNFSF13 | Hs00601664_g1 |
| EBI3 | Hs01057148_m1 |
| IL-27 | Hs00377366_m1 |
| IL-23A | Hs00900828_g1 |
| **Housekeeping Genes** |  |
| HPRT1 | Hs02800695_m1 |
| B2M | Hs00984230_m1 |
| GUSB | Hs00939627_m1 |
| TUBB | Hs00742828_s1 |
| GAPDH | Hs02758991_g1 |
